# Numerical Analysis of a SiN Digital Fourier Transform Spectrometer for a Non-Invasive Skin Cancer Biosensor

**DOI:** 10.3390/s25123792

**Published:** 2025-06-18

**Authors:** Miguel Ángel Nava Blanco, Gerardo Antonio Castañón Ávila

**Affiliations:** School of Engineering and Science, Tecnologico de Monterrey, Ave. Eugenio Garza Sada 2501, Monterrey 64849, Mexico; a00835327@tec.mx

**Keywords:** Raman spectroscopy, skin cancer, silicon integrated photonics

## Abstract

Early detection and continuous monitoring of diseases are critical to improving patient outcomes, treatment adherence, and diagnostic accuracy. Traditional melanoma diagnosis relies primarily on visual assessment and biopsy, with reported accuracies ranging from 50% to 90% and significant inter-observer variability. Among emerging diagnostic technologies, Raman spectroscopy has demonstrated considerable promise for non-invasive disease detection, particularly in early-stage skin cancer identification. A portable, real-time Raman spectroscopy system could significantly enhance diagnostic precision, reduce biopsy reliance, and expedite diagnosis. However, miniaturization of Raman spectrometers for portable use faces significant challenges, including weak signal intensity, fluorescence interference, and inherent trade-offs between spectral resolution and the signal-to-noise ratio. Recent advances in silicon photonics present promising solutions by facilitating efficient light collection, enhancing optical fields via high-index-contrast waveguides, and allowing compact integration of photonic components. This work introduces a numerical analysis of an integrated digital Fourier transform spectrometer implemented on a silicon-nitride (SiN) platform, specifically designed for Raman spectroscopy. The proposed system employs a switch-based digital Fourier transform spectrometer architecture coupled with a single optical power meter for detection. Utilizing a regularized regression method, we successfully reconstructed Raman spectra in the 800 cm^−1^ to 1800 cm^−1^ range, covering spectra of both benign and malignant skin lesions. Our results demonstrate the capability of the proposed system to effectively differentiate various skin cancer types, highlighting its feasibility as a non-invasive diagnostic sensor.

## 1. Introduction

Regular monitoring plays a critical role in managing various diseases and chronic conditions, significantly enhancing treatment adherence, enabling medication selection tailored to individual responses, and promoting healthier patient habits [1]. Recent years have witnessed the development of diverse technologies as potential solutions for portable and wearable health monitoring devices. Although many remain in prototype stages, preliminary results have demonstrated high promise, supporting their feasibility. Notable examples include cuffless blood-pressure monitors [2], non-invasive glucose monitors [3], virus detection systems [4], and on-site infection detection tools [5]. Early detection of skin cancer is particularly promising, as timely diagnosis can significantly reduce mortality rates [6].

Conventional clinical diagnosis of skin cancer typically involves visual inspection followed by invasive biopsy of suspicious lesions. Diagnostic accuracy for melanoma ranges between 50% and 90%, with up to one-third of cases exhibiting disagreement between dermatologist assessment and expert consensus [7]. Raman spectroscopy has emerged as a powerful non-invasive alternative capable of distinguishing cancerous cells from normal tissues [8,9]. A portable, real-time Raman spectroscopy system could serve as a complementary diagnostic tool, enhancing accuracy while reducing diagnostic time [10].

Silicon photonics has emerged as a leading platform for integrated photonic devices, capitalizing on well-established silicon complementary metal-oxide semiconductor (CMOS)-compatible fabrication processes [11,12]. This integration enables the cost-effective, high-yield, and scalable production of photonic systems suitable for high-volume applications [13]. While research in silicon photonics has traditionally focused on high-performance computing and optical communications, there is growing interest in its application for biomedical sensing and chronic disease monitoring [14].

Silicon photonics presents a promising approach for advancing portable Raman spectroscopy, enabling large detection volumes and field enhancement near high-index-contrast waveguides [15]. Furthermore, the minimal étendue of signals collected in single-mode waveguides facilitates ultra-compact integrated spectrometers with optimized spectral resolution [16]. Despite its potential, portable Raman spectroscopy technology faces fundamental and technical challenges [17]. A typical Raman spectrometer comprises three core components: a signal collection element, a dispersive element (e.g., a monochromator or diffraction grating) or a Fourier transform system, and a detector [18]. Raman signals are inherently weak and can be overwhelmed by fluorescence noise from organic and mineral samples [16]. Various strategies have been proposed to mitigate fluorescence interference, including mathematical algorithms [19], sequentially tunable laser schemes [19], and surface-enhanced Raman substrates that enhance Raman signals while suppressing fluorescence [20].

Resonator-based spectrometers, such as those employing ring resonators, offer compact footprints and high spectral resolution. However, their operation is confined to discrete resonance wavelengths, making them unsuitable for broadband spectral analysis as required in Raman spectroscopy of complex biological tissues [21]. Grating-based spectrometers—including arrayed waveguide gratings and diffractive optical systems—offer broader spectral coverage but suffer from intrinsic signal splitting across multiple output channels [22]. This reduces the optical power delivered to each detector, thereby degrading the signal-to-noise ratio (SNR), which is especially detrimental when measuring the weak signals typical of Raman scattering. In contrast, the switch-based digital Fourier transform spectrometer (sbDFTS) architecture utilizes a single output channel and benefits from the multiplex (Fellgett’s) advantage, resulting in improved SNR [18]. Additionally, its use of digitally tunable optical-path delays provides design flexibility and enables compact integration on platforms compatible with standard photonic foundry processes [23]. Another critical gap in Raman spectroscopy is the optimization of Fourier transform Raman spectrometers. Miniaturizing spectrometers for portable applications inherently involves trade-offs among device size, operational bandwidth, measurement speed, spectral resolution, dynamic range, signal intensity, and SNR [18]. Existing on-chip Fourier transform spectrometers generally operate in limited wavelength ranges, such as the silicon-based device developed by Kita et al. [24], covering 1550–1570 nm.

SiN-integrated Raman spectrometers have been used to analyze non-biological molecules. For instance, Raza et al. demonstrated an on-chip Raman sensor employing plasmonic nanotrenches in a SiN slot waveguide with a gold layer to detect 4-nitrophenol [25]. Dhakal et al. utilized a single-mode SiN strip waveguide to measure the Raman signature of pure isopropyl alcohol at 819 cm^−1^ [15]. Additionally, Turk et al. developed an integrated surface-enhanced Raman spectroscopy system based on a SiN nanoplasmonic slot waveguide, which successfully detected protease activity [26]. To the best of the authors’ knowledge, there are currently no demonstrations of integrated Raman spectrometers capable of measuring complex biological tissues such as skin. This work presents a simulation of a switch-based digital Fourier transform spectrometer (sbDFTS) optimized for Raman spectroscopy of skin tissues within the 800 cm^−1^ to 1800 cm^−1^ range (900–1000 nm) using an 830 nm, 250 mW excitation source. Spectral reconstruction is performed using the Basis Pursuit Denoising (BPDN) algorithm. Additionally, we generate synthetic Raman spectra of skin cancer phantoms, reconstruct them using the sbDFTS, and evaluate the reconstructed spectra against theoretical references [27] using the coefficient of determination. Our results highlight the system’s potential to differentiate among skin cancer types, underscoring its promise as a non-invasive diagnostic tool.

The primary innovation of our work lies in the application-specific optimization of the sbDFTS architecture for non-invasive Raman detection of skin cancer. While our design is inspired by the foundational work of Kita et al. [24], it introduces several key modifications tailored to the specific requirements of biomedical sensing. Notably, our system operates in a distinct spectral range (890 to 975 nm) optimized for capturing the Raman signature of skin, in contrast to the 1550 to 1570 nm range used in Kita’s work. Additionally, we employ a silicon nitride platform instead of a silicon-based one, allowing for improved performance in the near-infrared region relevant to our application. A custom-designed optical-path difference is implemented to enhance sensitivity to skin-specific spectral features. Furthermore, we integrate a specially engineered adiabatic coupler (AC) [28] optimized for the 800 nm to 1000 nm band, alongside a phase compensator and a digital path difference selector to correct for phase distortions introduced by the AC. These modifications collectively enable a more accurate and application-tuned spectral reconstruction suited for skin cancer diagnostics. Moreover, silicon nitride waveguide-based Raman systems have demonstrated significantly enhanced SNR and signal collection efficiency—outperforming conventional confocal Raman microscopes by up to three orders of magnitude, despite the presence of background noise from the waveguide core [29]. These advancements are paving the way for real-time monitoring of biological samples in integrated lab-on-chip platforms.

A key challenge in Raman spectroscopy remains efficient on-chip detection, particularly near-infrared detection, critical for various applications. Current near-infrared detectors often exhibit poor spectral responsivity, reliance on costly and hazardous III–V materials, or low responsivity [30]. Recent successful integration of III–V materials into silicon platforms via hybrid or monolithic methods has shown promising results [31,32]. Alternatively, germanium-based detectors offer a CMOS-compatible, cost-effective solution, achieving responsivity values exceeding 90% across a broad wavelength range (1.2–1.6 µm) at zero bias voltage and room temperature, extending into visible and ultraviolet spectra [33]. These advancements suggest a portable, wearable, non-invasive Raman monitoring system could soon become reality. In our case, we operate in the near-infrared window below 1 µm, and for this reason, we propose using an external germanium (Ge) photodetector, which offers improved sensitivity in the 890–975 nm range and can be coupled to the output of the sbDFTS. Although these Ge photodetectors are typically optimized for longer wavelengths (e.g., 1.2 µm and 1.6 µm), they still exhibit significant responsivity in the 900–1000 nm region, making them suitable for our application. Note that while integration of photodetectors is common in CMOS silicon photonics platforms, it is more challenging in silicon nitride (SiN) platforms due to the lack of compatible active devices such as photodetectors or optical amplifiers. This is one of the reasons we currently assume the use of an external semiconductor optical amplifier and a discrete Ge photodetector at the output of the sbDFTS. Nevertheless, the choice of SiN is motivated by its low background Raman emission, which is a critical advantage for Raman spectroscopy applications.

The remainder of this paper is structured as follows: Section 2 describes the materials and methods, Section 3 presents the results and discussion, Section 4 presents the future research, and Section 5 summarizes the conclusions.

## 2. Materials and Methods

The fundamental principle of a Fourier transform spectrometer (FTS) involves pre-processing the entire spectrum of the incident signal by tuning the optical-path difference (OPD) to obtain an interferogram. This interferogram is then reconstructed into the wavelength domain using appropriate algorithms [34]. FTSs leverage two key advantages: the Jacquinot advantage, which enables a higher amount of light to enter the system, and the multiplex advantage (Fellgett’s advantage), which enhances the SNR by simultaneously measuring all spectral frequencies.

According to the Rayleigh criterion, the spectral resolution (Δλ) is determined by [35]:

(1) $$\Delta \lambda =\frac{1}{N}\frac{\lambda^{2}}{n_{g}\Delta L}$$

where λ denotes the center wavelength, $n_{g}$ represents the group index of the waveguide, ΔL is the OPD, and ($N=2^{j}$) is the number of spectral channels.

To maximize sensing performance within the current SiN platform, we optimized our switch-based digital Fourier transform spectrometer (sbDFTS) architecture. Specifically, we conducted a systematic evaluation using different numbers of switching stages (j=6, 8, and 10), as well as a range of waveguide length differences—from 400 nm to 11.85 µm—to finely tune the optical-path difference (OPD). After extensive simulation and testing, the configuration with j=8 switches (N=256 spectral channels) and an OPD of 400 nm consistently provided the best balance between spectral resolution, signal-to-noise ratio, and system compactness. These findings were critical for achieving acceptable spectral reconstructions, despite the modest performance of currently available detectors in our target wavelength range.

### 2.1. Switch-Based Digital Fourier Transform Spectrometer

**Figure 1:**
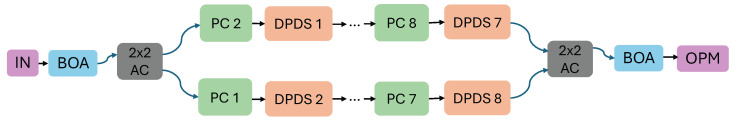
Diagram illustrating the design concept of the switch-based digital Fourier transform spectrometer sbDFTS.

The sbDFTS design is inspired by the work of Kita et al. [24]. The schematic, shown in Figure 1, illustrates that the sbDFTS consists of an input, where the Raman spectrum is injected, followed by a booster optical amplifier (BOA). A 2 × 2 3 db adiabatic coupler (AC), developed by [28], splits the incoming signal into two separate paths. The two arms of the interferometer contain phase compensators (PCs) and digital path difference selectors (DPDS). Finally, the output of the last AC is connected to a BOA, and the output to the optical power meter (OPM). We assume the BOA used is the Thorlabs model BOA930S, with a signal gain of 30 dB, a noise figure of 8.5 dB, a noise center frequency of 935 nm, and a noise bandwidth of 37 nm. Additionally, the broadband phase shifter presented in [36] is included in the design, featuring an insertion loss of 0.711 dB. The SiN waveguide loss is assumed to be 0.8 dB/cm, extrapolated from NanoSOI Design Center data for the wavelength range under consideration. The laser used to stimulate the Raman spectrum and the BOA are external devices. However, techniques such as monolithic integration have been explored, demonstrating promising results [31,32]. Consequently, the hybrid or monolytical integration of lasers and amplifiers on the SiN platform is considered only a matter of time [13]. Other components, such as thermo-optic phase shifters, are already available. Nevertheless, promising broadband phase shifters operating in the visible and near-infrared ranges have also been reported [36].

**Figure 2:**
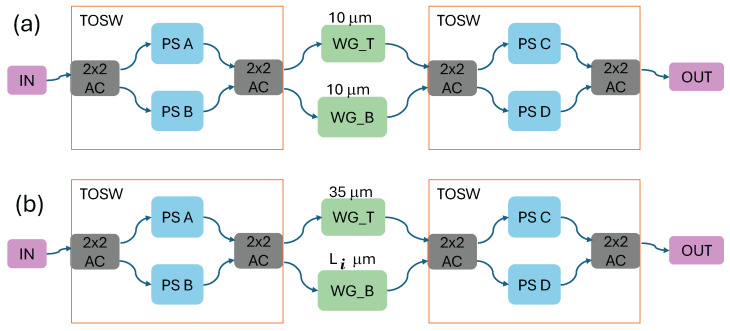
Diagram showing the detailed design of the (**a**) phase compensator PC and (**b**) digital path-difference selector DPDS used in the sbDFTS.

The PC and DPDS structures are presented in Figure 2a and Figure 2b, respectively. The PCs are used to compensate for phase shift variations introduced by the realistic adiabatic couplers and the DPDS. We observed that when digitally selecting different optical-path differences (OPDs), the signals in both arms must pass through the same number of components. This necessitates the use of phase compensators.

The PC structures consist of a thermo-optic switch (TOSW) comprising an adiabatic coupler (AC) that splits the signal, two thermo-optic phase shifters (PSs), and a second AC that recombines the signals. In all PC elements, the top and bottom waveguide lengths are set to 10µm. Following the waveguides, another TOSW is connected to the output. The DPDS structures are similar to the PCs, but with a fixed top waveguide length of 35µm, while the bottom waveguide lengths vary as follows: $L_{1}=34.6\;µ$m, $L_{2}=35.8\;µ$m, $L_{3}=33.4\;µ$m, $L_{4}=38.2\;µ$m, $L_{5}=28.6\;µ$m, $L_{6}=47.8\;µ$m, $L_{7}=9.4\;µ$m, and $L_{8}\;=\;86.2\;µ$m. The OPD can be adjusted by toggling the switches in the sbDFTS. The DPDS arrangement, defined as $(DPDS_{8},\;DPDS_{7},\;DPDS_{6},\;DPDS_{5},DPDS_{4},\;DPDS_{3},\;DPDS_{2}$, and $DPDS_{1})$, determines the configuration, with the OPD given by the equation $\Delta L=2^{k}\times 400$ nm, where *k* represents the configuration value.

The waveguide design parameters comply with the manufacturability constraints specified by the NanoSOI Design Center. These constraints include a minimum feature size of 120 nm, a SiN device layer thickness of 400 nm, a buried thermal oxide layer of 4.5µm, and a plasma-enhanced chemical vapor deposition (PECVD) oxide cladding of 3µm. Additionally, experimental refractive index data for SiN and SiO_2_ were provided by NanoSOI. Additionally, wherever waveguides of different widths are connected, optimized linear tapers are used, each with a length of 10µm. These tapers were designed and simulated using the Finite-Difference Time-Domain (FDTD) tool from Ansys Lumerical 2024 to obtain the S-parameters while adhering to the NanoSOI design constraints.

### 2.2. Ideal Fourier Transform Spectrometer

To validate the performance of the sbDFTS, we designed an ideal Fourier transform spectrometer (iFTS) based on a single Mach–Zehnder interferometer (MZI). Numerical analysis was performed using ANSYS Lumerical Interconnect 2024. The MZI consists of an ideal 3 dB Y-junction that splits the incoming signal into two separate paths, two SiN strip waveguides of lengths $L_{t}$ and $L_{b}$, and a second ideal 3 db Y-junction that recombines the optical signals, where interference occurs. Finally, the output of the last Y-junction in each interferometer is connected to an optical power meter (OPM).

Since we are considering 256 channels, this is equivalent to using a single unbalanced MZI in 256 different configurations, where each configuration receives the same Raman spectrum as input. We constructed two waveguide length vectors, each containing 16 elements: one for the top arm, denoted as $\left(L_{t_{i}}\right)$, and the other for the bottom arm, denoted as $\left(L_{b_{j}}\right)$. The subscripts allow systematic combinations of different arm lengths. To generate the 256 MZI configurations, each element of the vector $\left(L_{t_{i}}\right)$ was combined with every element of the vector $\left(L_{b_{j}}\right)$. The specific values used were the following: $L_{t_{i}}$ = 140, 140.8, 143.2, 144, 152.8, 153.6, 156, 156.8, 191.2, 192, 194.4, 195.2, 204, 204.8, 207.2, 208 µm; $L_{b_{j}}$ = 140, 139.6, 138.4, 138, 133.6, 133.2, 132, 131.6, 114.4, 114, 112.8, 112.4, 108, 107.6, 106.4, 106 µm. These values were carefully chosen to ensure that the optical-path differences (OPDs) are multiples of 400nm, thereby improving the spectral resolution. Furthermore, these OPD combinations correspond to those available in an eight-stage sbDFTS system.

### 2.3. Spectrum Reconstruction Method

The problem of spectral reconstruction can be formulated as a system of linear equations:

(2) $$\vec{y}=A\vec{x},$$

where $\vec{y}$ is the observation vector, A is the calibration matrix, and $\vec{x}$ is the spectrum to be reconstructed. In this work, the linear system is an underdetermined system, meaning it has more unknowns than equations, and therefore admits infinitely many solutions. So, more constraints are needed if we want to find a suitable solution and, therefore, a suitable spectrum. For the spectrum reconstruction injected into the sbDFTS, we employ the BPDN algorithm, which solves the following mathematical optimization problem:

(3) $$\min_{\vec{x}}\left(\frac{1}{2}\left|\right|\vec{y}-A\vec{x}{\left|\right|}_{2}^{2}+\lambda ||\vec{x}{\left|\right|}_{1}\right).$$

The above optimization problem constrains the possible solutions, where the Euclidian norm ($\left|\right|\cdot {\left|\right|}_{2}$) measures the magnitude of the error between the model predictions and observed data, and also ensures data fidelity, that is, the reconstructed signal closely matches to the observed measurements. Meanwhile, the $L_{1}$ norm ($\left|\right|\cdot {\left|\right|}_{1}$) measures the sum of the absolute values of the solution $\vec{x}$, and it includes the sparsity constraint in the model, that is, most entries of $\vec{x}$ are zero or near zero. This is particularly useful in scenarios like compressed sensing or Raman spectrum reconstruction, where the true signal consists of a few significant features or peaks. In this optimization problem, λ enforces a trade-off constraint between sparsity and fidelity in the reconstruction of the spectrum vector $\vec{x}$. A small value of λ places greater emphasis on fitting the observed data $\vec{y}$, which may also lead to overfitting, particularly in noisy environments. A large value of λ enforces sparsity but risks underfitting, shadowing important spectral features [24]. In this work, the columns of the calibration matrix A consist of the interferograms measured by the iFTS, using a continuous wave laser (CWL) with a power of 100 mW, a full-width at half-maximum (FWHM) of 0.5 nm, and a wavelength range from 890 nm to 975 nm in steps of 0.5 nm. To systematically determine an optimal value of λ, we employed a 10-fold cross-validation strategy, a standard approach in regularized regression. The process was as follows:

1. The available dataset was divided into 10 equally sized folds.
2. For each candidate value of λ, the algorithm was trained on nine folds and tested on the remaining one.
3. This process was repeated 10 times, rotating the validation fold each time.
4. The Mean Squared Error (MSE) between the reconstructed spectrum and the known ground truth was computed for each fold.
5. The λ value that minimized the average MSE error across all folds was selected as optimal.

**Figure 3:**
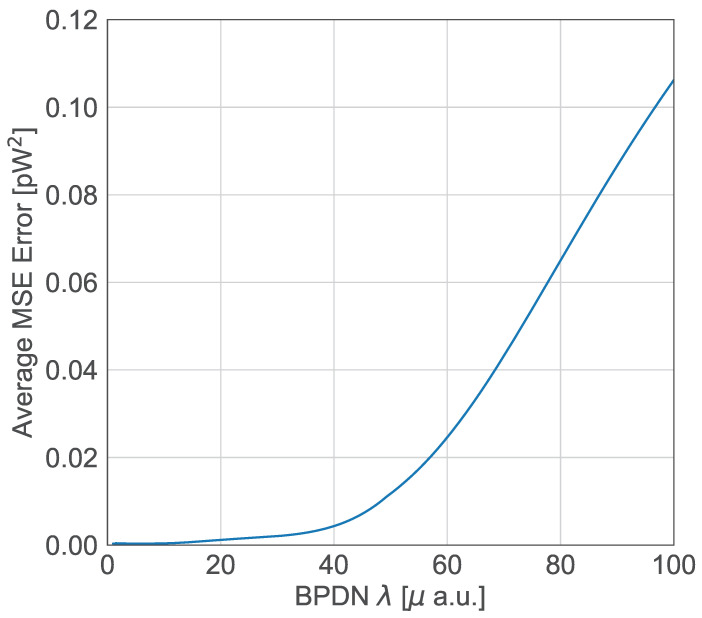
Average MSE error vs. the regularization parameter λ in the melanoma spectrum reconstruction. The optimal value λ = 1.365 × 10^−6^ is relatively low due to the broad Lorentzian peaks present in the signal. Subtracting the skin fluorescence background from the melanoma spectrum is a crucial preprocessing step—without it, the optimal λ value would be even smaller, and the BPDN algorithm would likely fail to reconstruct the Raman spectrum accurately.

This approach ensures that the selected λ generalizes well to unseen data and avoids overfitting. The cross-validation was performed over a logarithmic sweep of λ values, typically ranging from $10^{-6}$ to $10^{6}$, allowing the algorithm to explore a wide range of sparsity-enforcing regimes. In practice, we found that the optimal λ varied depending on the complexity of the input spectrum. For spectra with sharp and isolated peaks, higher sparsity (λ) was preferable. In contrast, for spectra containing overlapping features or broad background signals (such as fluorescence), a low λ produces a low MSE error as observed in Figure 3, and therefore better reconstructions. By using cross-validation, we avoided arbitrary or heuristic choices of the regularization parameter and instead grounded the selection in quantitative performance metrics, enhancing the robustness and reproducibility of the reconstruction pipeline.

### 2.4. Phantom Cancer Raman Spectra

For the development of several phantom cancer Raman spectra, we used the work done by Lui et al. [27]. We modeled the phantom Raman spectra of melanoma (MEL), basal cell carcinoma (BCC), squamous cell carcinoma (SCC), actinic keratosis (AK), atypical nevi (AN) and seborrheic keratosis (SK), as a series of Lorentzian peaks:

(4) $$y_{R}=\sum_{i=1}^{N}\frac{2A_{i}}{\pi }\frac{\omega_{i}}{4{\left(r-r_{i}\right)}^{2}+\omega_{i}^{2}},$$

where $r_{i}$ denotes the peak position, $A_{i}$ is the total area under the curve from the baseline, and $\omega_{i}$ is the bandwidth of the peak at the FWHM. The coefficients are given in Table 1. The fluorescense skin signal was modeled as

(5) $$\begin{array}{cc} y_{f}= & 382.2-9.06\times 10^{-1}r+1.560\times 10^{-3}r^{2}-1.443\times 10^{-6}r^{3} \end{array}$$

(6) $$\begin{array}{cc} \; & +6.721\times 10^{-10}r^{4}-1.240\times 10^{-13}r^{5}, \end{array}$$

where *r* represents the Raman shift.

**Figure 4:**
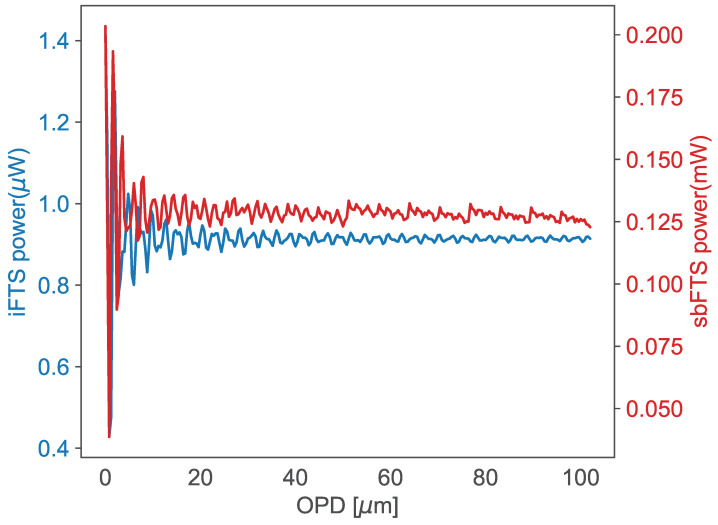
Interferograms of melanoma made by the iFTS and by the sbDFTS. The different scales in power are due to the inclusion of optical amplifiers in the sbDFTS.

These spectra were injected into the sbDFTS, and the resulting interferograms were measured (see Figure 4). A measurement consists of injecting the Raman spectrum into the input port of the sbDFTS. The resulting output power *P* measured by the OPM is recorded as a pair (ΔL, *P*) for all ΔL. To reconstruct the Lorentzian peaks, we first measure the fluorescence signal given by $y_{f}$, then the Raman skin signal ($y_{R}+y_{f}$). The data obtained from the fluorescence signal are subtracted from the data obtained from the Raman skin signal, and the BPDN algorithm is applied to the resulting difference.

## 3. Results and Discussion

**Figure 5:**
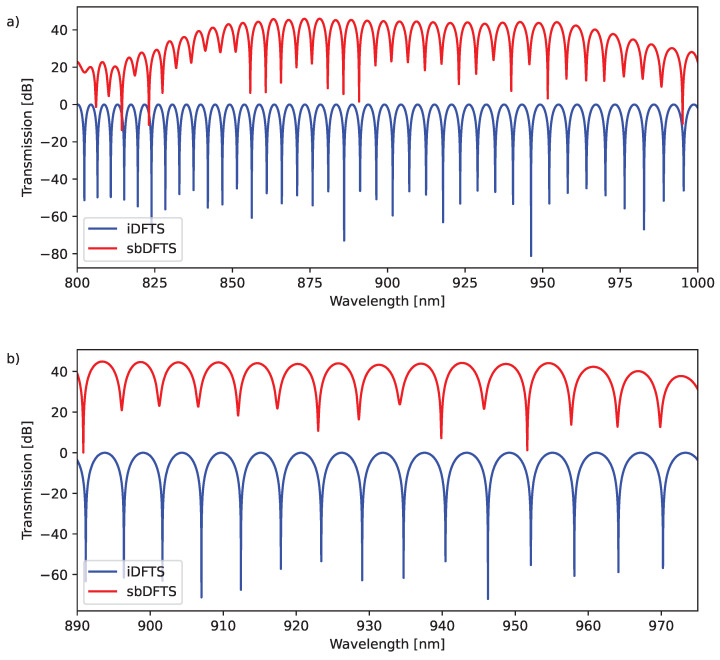
Comparison of the FSR for the (11001101) configuration of the sbDFTS and ΔL=71.6µm for the iFTS. We get the values p=0.7418, t=0.3324, and $t_{\mathrm{critic}}=2.0369$. (**a**) Transmission for range 800–1000 nm, and (**b**) for range 890–975 nm.

The behavior of the sbDFTS is expected to differ from that of the iFTS due to the inclusion of realistic components and optical amplifiers. However, to enable a fair comparison between both spectrometers, we perform a Free Spectral Range (FSR) analysis using the same OPD for both systems. Figure 5a reveals similar trends in the fluctuations of the transmission function. However, we limit the wavelength range to 890–975 nm shown in Figure 5b, where transmission is more uniform, and employ a *t*-test to assess the statistical significance of the differences. For configurations where the *t*-test is applicable (configurations 24 to 255), the results do not reject the null hypothesis at a 5% significance level. However, for configurations below 24, only a single destructive interference peak is observed, preventing any statistically significant conclusions.

Table 2 presents the $R^{2}$ coefficients of determination comparing the model based on Lorentzian peaks (simulated data) with the sbDFTS-reconstructed spectra (experimental data). The highest coefficient of determination ($R^{2}$) for the experimental data of AK corresponds to the simulated SCC data ($R^{2}=0.875$). For the experimental AN data, the best match is with the simulated AN data ($R^{2}=0.867$). The highest $R^{2}$ for BCC is found with the simulated SCC data ($R^{2}=0.867$), while for MEL, the best match is with the simulated MEL data ($R^{2}=0.867$). For SCC, the experimental data best matches the simulated SCC data ($R^{2}=0.863$), and for SK, the experimental data aligns with the simulated SK data ($R^{2}=0.871$). This indicates that pigmented lesions (MEL, AN, SK), which have higher Raman signals, are better recognized by the sbDFTS than nonpigmented skin conditions (BCC and SCC, and AK).

**Figure 6:**
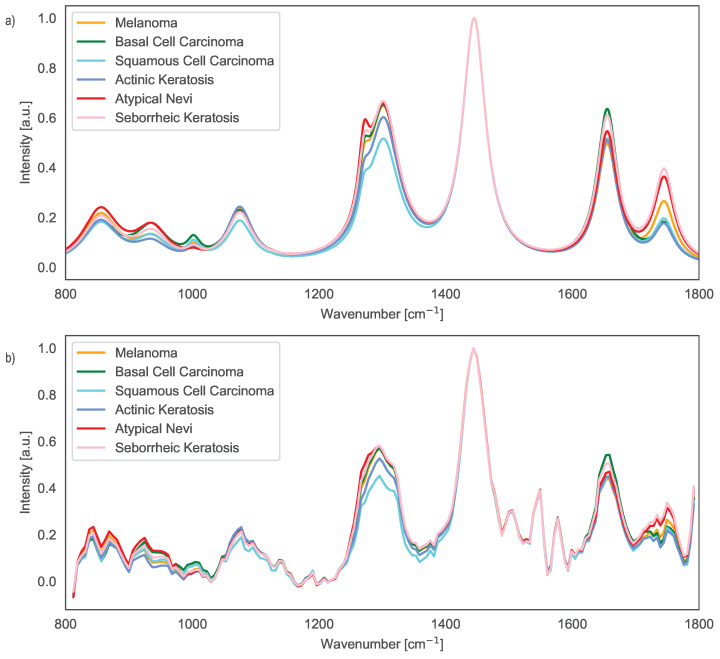
(**a**) Normalized simulated cancer Lorentzian peaks and (**b**) normalized reconstructed spectrum from experimental data. Peaks at 1271 cm^−1^ and 1302 cm^−1^ are merged due the moderate spectral resolution of the sbDFTS (46 cm^−1^), and the peak at 1745 cm^−1^ is less pronounced due the optical 3 dB bandwidth of the BOA930S.

Figure 6a shows the normalized simulated cancer Lorentzian peaks and Figure 6b shows the normalized reconstructed spectrum from experimental data. Observations from Figure 6b indicate that the reconstructed spectra are noisy. The sbDFTS struggles to distinguish the peaks at $1271\;{\mathrm{cm}}^{-1}$ and $1301\;{\mathrm{cm}}^{-1}$, and there are artifacts observed around $1445\;{\mathrm{cm}}^{-1}$ and $1655\;{\mathrm{cm}}^{-1}$. The peak at $1745\;{\mathrm{cm}}^{-1}$ is also not well-reconstructed. The theoretical spectral resolution of the sbDFTS is approximately Δλ=4nm, which corresponds to about $46\;{\mathrm{cm}}^{-1}$ in wavenumber. This inherently limits the system’s capability to resolve spectral features separated by less than 4 nm. Consequently, peaks at $1271\;{\mathrm{cm}}^{-1}$ and $1302\;{\mathrm{cm}}^{-1}$ in Figure 6b appear merged due to the moderate spectral resolution of the sbDFTS (∼46 ${\mathrm{cm}}^{-1}$). Additionally, the peak at $1745\;{\mathrm{cm}}^{-1}$ appears less pronounced in the reconstructed spectra, primarily due to increased noise and reduced optical amplification from the BOA930S at the spectral edges.

We define sensitivity as the ability of the device to detect weak optical signals. To quantify this, we used the signal-to-noise ratio (SNR), since a more sensitive spectrometer is expected to distinguish weak signals from the noise background. In this context, we adopted a definition of SNR by Horiba [37], expressed as the difference of peak signal and background signal, divided by the root mean square (RMS) value of the noise on the background signal: SNR = $10log_{10}\left(\left(S_{p}-S_{b}\right)/N_{RMS}\right)$ where $S_{p}$ is the peak signal, $S_{b}$ is the background signal, and $N_{RMS}$ is the RMS value of the noise on the background signal.

**Figure 7:**
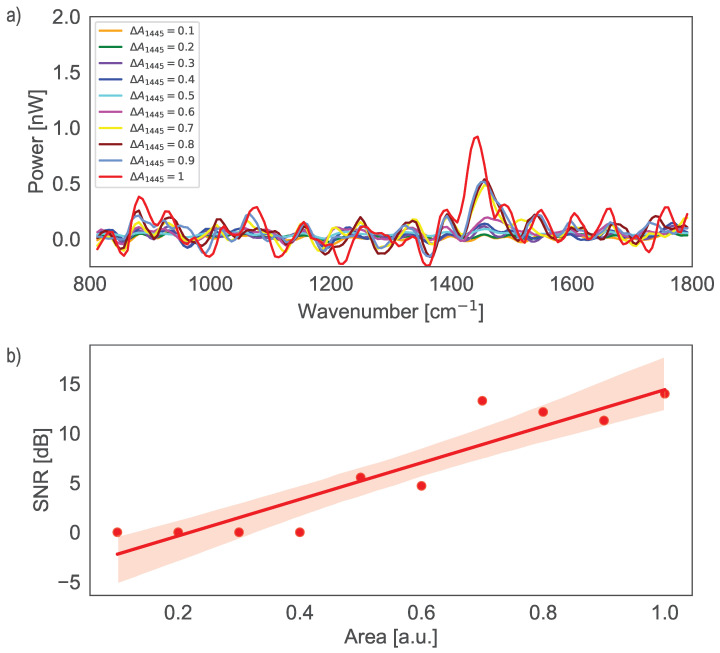
(**a**) Power (nW) ΔG vs. λ cm^−1^ for various values of $A_{1445}$, showing the changes in the Raman spectrum at 1445 cm^−1^; (**b**) SNR dispersion vs. Area [a.u.] for various values of $A_{1445}$.

Figure 7a illustrates that when $A_{1445}\geq 0.5$ or, equivalently, when the power difference ΔP between the original Raman skin spectrum, $G_{0}$, at 1445 cm^−1^ with $A_{1445}$ = 0.1 and the modified spectrum, $G_{i}$, exceeds 0.075 nW, the variation in the peak becomes distinguishable. The difference between these two spectra is denoted as ΔG, obtained by subtracting $G_{0}$ from $G_{i}$. As shown in Figure 7b, the signal level exceeds the noise level when the SNR is greater than 0 dB. This condition is satisfied when $A_{1445}=0.5$ or, equivalently, when the power difference ΔP exceeds 0.075 nW.

While this analysis demonstrates that these variations can be detected under controlled conditions, further research using real optical sensors is necessary to validate whether such differences can be measured in practical applications and how the values of $A_{1445}$ correlate with real tissue samples.

The primary sources of noise in the sbDFTS originate from the non-ideal behavior of the adiabatic 3 db coupler. This coupler operates at the edge of the adiabatic regime, resulting in a wavelength-dependent and non-uniform power splitting ratio. Additionally, the phase shifters introduce further potential error, as each incorporates the same non-ideal adiabatic coupler. In our simulations, we assume a waveguide propagation loss of approximately 0.8 dB/cm. To compensate for these losses, optical amplifiers are placed at both the input and output of the device, each with a noise figure of 8.5 dB, a center wavelength of 935 nm, and an optical 3 dB bandwidth of 37 nm. Furthermore, linear tapers are implemented throughout the design to ensure smooth transitions between waveguides of different widths, thereby minimizing coupling losses.

Raman artifacts may arise due to the interaction between system noise and the reconstruction algorithm. In particular, the BPDN algorithm is susceptible to detecting false or non-existent peaks when exposed to noisy or broadband signals. This occurs because BPDN attempts to reconstruct the sparsest possible signal consistent with the measured interferogram, and in the presence of noise or low SNR, it may misinterpret random fluctuations as valid spectral features.

This analysis shows the feasibility of skin cancer detection using the sbDFTS. However, as there are no distinctive Raman peaks or bands that can be uniquely assigned to a specific skin pathology, real data and sophisticated statistical methods as in [10] shall be used to give an accurate diagnosis. These methods in [10] have demonstrated confidence intervals ranging from 95% to 99%. Our simulated device has shown promising results in reconstructing various cancer spectra. As a future research, experimental validation will be the next step to assess the potential of the sbDFTS as a non-invasive cancer biosensor.

The synthetic Raman spectra used in this study were constructed based on in vivo tissue data reported by Lui et al. [27]. While the spectra in that paper had already undergone preprocessing steps—including fluorescence background subtraction, smoothing, and normalization—we opted to reconstruct synthetic Raman spectra using Lorentzian peak fitting based on the digitized spectral features. Additionally, the Raman skin fluorescence signal was modeled as a fifth-order polynomial. This approach allowed us to generate controlled and repeatable test signals that mimic the main Raman signatures of different skin cancer types, while preserving the spectral structure described in the original work [27]. Importantly, we did not use the preprocessed spectra as-is; instead, we modeled the key peaks analytically, allowing us to isolate the performance of the spectrometer and reconstruction algorithm from unpredictable noise and variability. Nonetheless, these synthetic spectra serve as a valid and controlled testbed for evaluating the spectral reconstruction capabilities of the proposed sbDFTS method. Since our current input data are simulated and not derived from labeled patient samples, statistical performance metrics such as principal component analysis (PCA) or partial least squares regression (PLSR) are not yet applicable. We view this work as an important preliminary step toward future experimental validation with real tissue samples, which is currently under development.

The primary objective of this study was to demonstrate the capability of sbDFTS to reconstruct complex Raman spectra, and to explore its potential utility in biomedical applications such as cancer diagnostics. While our synthetic spectra are grounded in experimental data from real tissue samples, it is important to note that specific and universally accepted Raman biomarkers for distinct skin pathologies remain poorly defined in the current literature. Therefore, our reconstructed spectra do not aim to represent definitive diagnostic signatures at this stage, but rather to showcase the method’s potential for accurate spectral reconstruction. We believe that as more biomolecular signatures become established through future clinical studies, methods like sbDFTS will play a valuable role in enhancing diagnostic reliability and interpretability.

## 4. Future Research

While this study primarily focuses on demonstrating the feasibility of using sbDFTS for Raman spectral reconstruction, experimental validation is required as future research. Next steps include fabricating a prototype sbDFTS on a silicon nitride (SiN) platform using commercial foundry services (e.g., NanoSOI), integrating it with a tunable near-infrared laser and a fiber-coupled Raman probe, and testing the complete system using skin phantoms and/or excised biological tissues. Is important also to address practical challenges such as optical alignment, background noise suppression, and robust sample handling, all of which are critical for reliable spectral acquisition in a diagnostic setting. These efforts will pave the way for future in vivo validation and the eventual integration of the system into a portable, non-invasive diagnostic device.

Moreover, future research should prioritize the acquisition and analysis of real patient Raman spectra to rigorously evaluate the diagnostic performance of the sbDFTS method. Comparative studies involving established diagnostic tools—such as traditional spectroscopic classifiers or machine learning-based diagnostic frameworks—would provide critical benchmarks for assessing the added value of sbDFTS in clinical settings. Furthermore, integrating statistical validation metrics like accuracy, sensitivity, and specificity will be essential to quantitatively substantiate the method’s utility. In parallel, advanced data mining techniques could be employed to uncover latent diagnostic features within the reconstructed spectra, potentially revealing new biomarkers or enhancing disease differentiation. These efforts will not only strengthen the clinical relevance of the method but also help position sbDFTS within the broader landscape of biomedical diagnostic technologies.

## 5. Conclusions

In this work, we demonstrated the application of the sbDFTS combined with the BPDN algorithm for Raman spectrum reconstruction, specifically targeting complex spectra associated with skin cancer. Although the system was not able to accurately categorize all reconstructed spectra, promising results were observed through a close resemblance between reconstructed and theoretical spectra, even in the presence of noise and artifacts. Such robustness highlights the potential of the sbDFTS as a tool for early cancer diagnosis, emphasizing its capability to handle real-world complexities inherent in biological samples. However, further validation through experimental implementations using fully integrated photonic setups, realistic biological samples, and comprehensive statistical analyses remains essential to rigorously assess and enhance its diagnostic accuracy and practical applicability.

As an initial proof-of-concept, the sbDFTS system demonstrates substantial promise for Raman spectroscopy, effectively reconstructing intricate spectral features relevant to biological tissues. The successful integration of sbDFTS with the BPDN algorithm underscores its potential for significant advancements in diagnostic methodologies, particularly for early and accurate cancer detection. Future research will focus on optimizing device parameters, including improving spectral resolution, noise reduction strategies, and expanding experimental validation. Additionally, exploring the system’s applicability to a broader range of biomarkers and diseases beyond skin cancer will be critical in establishing sbDFTS as a versatile, non-invasive diagnostic platform for diverse biomedical applications.

## Figures and Tables

**Table 1:** Parameters used in the construction of the various Lorentzian peaks for each cancer type. The coefficients were chosen arbitrarily, but the shape of the spectra was designed to resemble in vivo tissue. Center of the Raman shift wavenumber $r_{0}$ (cm^−1^), FWHM $\omega_{0}$ (cm^−1^), and area under the Lorentzian peak $A_{i}$ [a.u.].

		MEL	BCC	SCC	AK	AN	SK
$r_{0}$	$\omega_{0}$	$A_{i}$	$A_{i}$	$A_{i}$	$A_{i}$	$A_{i}$	$A_{i}$
855	68	58	44	53.5	57	58	47
936	62	23.7	35	29	22.3	32.5	26
1002	24	4.5	6.5	7	4	1.5	3
1075	48	43	39	38	51	37	35
1125	10	0.2	0.2	0.2	0.2	0.2	0.2
1271	20	15	16	12	13	21.2	16
1302	60	155	146	137	162	142.3	138
1445	44	182	171	205	205	167	158
1655	39	78.6	96.3	92.5	91.5	79	84
1745	39	39.4	23	31	27.5	51	52.5

**Table 2:** $R^{2}$ coefficients of determination between simulated data and experimental data.

	AK_s	AN_s	BCC_s	MEL_s	SCC_s	SK_s
AK_e	0.8671	0.8062	0.8184	0.8486	0.8755	0.8046
AN_e	0.8423	0.8674	0.8329	0.8624	0.8394	0.8625
BCC_e	0.8664	0.8507	0.8664	0.8642	0.8675	0.8579
MEL_e	0.8658	0.8436	0.8316	0.8670	0.8648	0.8394
SCC_e	0.8247	0.7465	0.7642	0.7950	0.8625	0.7439
SK_e	0.8422	0.8639	0.8348	0.8596	0.8424	0.8709

## Data Availability

Data underlying the results presented in this paper are not publicly available at this time but may be obtained from the authors upon reasonable request.
